# Large-Scale Combat Operation Education and Training Needs: Implications for Military and Civilian Medical Education

**DOI:** 10.5811/westjem.43557

**Published:** 2025-09-01

**Authors:** Rebekah Cole, Kiia Crawford, Makinna Farrell, Leslie Vojta, Sherri L. Rudinsky

**Affiliations:** * Uniformed Services University, Department of Military and Emergency Medicine, Bethesda, Maryland; † Uniformed Services University, Department of Health Professions Education, Bethesda, Maryland; ‡ Uniformed Services University, School of Medicine, Bethesda, Maryland

## Abstract

**Introduction:**

Future large-scale combat operations (LSCO) with adversaries such as Russia or China are predicted to present unique challenges for medical personnel, including high casualty rates, limited resources, and austere environments. While traditionally associated with military conflict, the anticipated scale of future LSCO may overwhelm military medical systems, requiring civilian physicians to support wartime care or manage surges in casualties on the home front. Effective training early on is, therefore, critical to prepare both military and civilian physicians for these unique and demanding conditions.

**Methods:**

We used interpretive phenomenological analysis in this qualitative study to explore key competencies needed for LSCO medical readiness. The first and seniors author interviewed 27 military physicians (active duty, retired, and reservists) and one military chaplain with extensive operational medical experience in the fall of 2023 and fall of 2024. We analyzed transcripts to identify recurring themes. Data analysis was conducted by a diverse and experienced research team.

**Results:**

Five key themes emerged as essential for LSCO-focused medical training: 1) problem-solving in resource-limited environments, emphasizing critical thinking and improvisation; 2) ethical and emotional resilience, addressing psychological and moral challenges; 3) adaptive leadership, highlighting decision-making in high-stress settings; 4) mastery of core medical skills, ensuring competency in essential procedures; and 5) cultural competence and interoperability, supporting effective collaboration across military-civilian teams.

**Conclusion:**

The competencies identified in this study are relevant to both military and civilian physicians who may be called upon to provide care during large-scale combat operations. Medical education must proactively incorporate these themes to ensure readiness across both sectors. Strengthening military-civilian collaboration in training and curriculum development will enhance national preparedness for future conflicts.

## INTRODUCTION

The United States Department of Defense predicts that future large-scale combat operations (LSCO) with peer/near-peer adversaries such as Russia or China will take place in volatile, uncertain, complex, and ambiguous environments within the next 5–10 years.^1^ These imminent conflicts may unfold in austere combat settings with restricted resupply, air evacuation, and access to surgical care.^2^ As a result, the death toll of LSCO is predicted to match that of World War II, where an estimated 15 million service members and 38 million civilians were killed, with many more wounded.^3^ Similarly, recent regional conflicts, such as those in Israel and Ukraine, serve as stark reminders of the significant impact and stresses such conflicts place on both military and civilian medical infrastructure and communities.^4^ Physicians near war zones will face challenges related to high patient volumes, severe injuries, and limited resources, demonstrating the broad applicability of these concepts across civilian and military medical contexts.^5^

Both military and civilian medicine will need to overcome the formidable challenges of LSCO.^3^ Military physicians will lead healthcare teams in austere environments with limited resources, often requiring prolonged casualty care when evacuation delays stretch beyond the previous standard “golden hour.”^6^ Similarly, civilian physicians may be called upon to support wartime care and/or manage surges in casualties on the homefront.^3^ These realities necessitate a re-evaluation of how physicians—both military and civilian—are trained for war-time crisis response at home and abroad, as these unique conditions merit distinct educational considerations.

To ensure readiness, physicians must develop a multifaceted skill set to lead healthcare teams during LSCO. For example, they may be called to serve as leaders, trainers, and advisors who advocate for the health and welfare of their units.^7^ They also may be called to assume leadership roles in coordinating care across multiple agencies and institutions.^8^ Therefore, training physicians in leadership, adaptability, and resource management is essential for ensuring both mission success and improved patient outcomes. Medical students, especially, can benefit from this training as they represent a formative stage where foundational professional identity, leadership capacity, and adaptability are being developed. Early exposure to the realities of LSCOs can help frame future clinical decision-making, resilience, and operational readiness in unique military environments.^9^,^10^,^11^,^12^

Despite the urgent need for LSCO preparedness, there is little precedent or prior research on how to best equip medical students for such operations, as the United States has not engaged in such large-scale peer/near-peer warfare since World War II. Past research has shown that preparation in medical school increases physicians’ readiness and leadership abilities in volatile wartime environments.^13^ Current educational programs focus on leadership, problem-solving, and resilience in the context of natural disasters and mass casualty events; however, LSCO are expected to involve extensive prolonged casualty care, limited evacuation capabilities, degraded communications, and heightened moral and logistical complexity.^14^,^15^,^16^,^17^,^18^,^19^ As a result, no existing programs are specifically designed to address the unique uncertainty, scale, and ethical challenges posed by LSCO, which are factors not typically covered in standard disaster medicine curricula. To begin addressing this gap, we conducted a qualitative study with experienced military medical educators to explore their perceptions of what education and training are needed during medical school to prepare future military and civilian physicians for LSCO.

**Population Health Research Capsule**

What do we already know about this issue?

*Medical readiness for large-scale combat operations (LSCO) is underdeveloped in both military and civilian medical education*.

What was the research question?

*What competencies are needed to prepare physicians for future LSCO?*

What was the major finding of the study?

*Key themes emerged as vital for LSCO medical training: critical thinking and improvisational skills, emotional resilience, adaptive leadership, and cultural competence*.

How does this improve population health?

*Identifying LSCO training needs helps prepare physicians for crisis care, improving survival and ethical decision-making in high-stakes environments*.

## METHODS

We chose an interpretive phenomenological analysis (IPA) to guide our study, acknowledging our role as researchers, military physicians, and educators and the influence these roles had on our interaction with our participants and our interpretation of the data.^20^ The goal of IPA is to depict how the participants perceive a particular phenomenon. In our study we aimed to describe in depth how our participants made sense of military medical education and training in preparation for future large-scale conflicts.

The participants in our study were 27 active-duty, retired, and reserve military physicians and one military chaplain, convenience sampled from available faculty attending military medicine operations. All our participants had significant experience with military medicine in the operational environment. (See Appendix A for participant demographics.) The participants were selected for their military backgrounds and experience within military medical education. All participants had deployed in support of combat operations, primarily in Iraq and/or Afghanistan.

We also included one military chaplain in our study because of their extensive operational experience supporting medical teams during combat deployments and their unique insight into the ethical, emotional, and moral challenges faced by healthcare personnel in LSCO. We first recruited participants via email and in person while they were serving as faculty at our university’s capstone Medical Field Practicum during the fall of 2023. All participants volunteered for the study. None were recruited or coerced by a military commanding officer. The participants received an institutional review board (IRB)-approved information sheet describing the study goals and research processes during participant recruitment. The study goals outlined on the information sheet provided to participants were 1) to explore how experienced military medical educators perceive the training needs of future physicians in the context of large-scale combat operations, and 2) to identify key educational priorities that could inform medical school curriculum development.

After analyzing our data and determining that saturation had not been reached, we recruited more participants during the fall of 2024 at the same exercise.^21^, ^22^ The first and senior authors conducted audio- recorded in-person, semi-structured interviews with each of these participants. No field notes were taken during the interview process, and no one else was present during the interviews. The first author is an experienced qualitative researcher with a doctoral degree. The senior author is a medical doctor and has completed graduate coursework in qualitative research methodology. The interviews averaged 43 minutes each. All participants were interviewed using the same semi-structured interview guide, which was developed based on our research questions and reviewed by our team prior to data collection. The same interview protocol was used across both recruitment cycles (Fall 2023 and Fall 2024), and all interviews were conducted in person by either the first or senior author. While follow-up questions were asked during individual interviews, these were used to clarify or deepen participant responses and were not part of a separate, standardized second interview.^20^

We then transcribed each of these interviews using an automated transcription service. To ensure the credibility of our data, we emailed the transcripts back to the participants, asking for their feedback on their interviews, which is a practice known as member checking. This study was approved by the IRB at the authors’ university (DBS.2021.248). No participants refused to participate or dropped out of the study.

Following the steps of IPA, our research team first reviewed Participant 1’s transcripts, noting important training aspects throughout.^23^, ^24^, ^25^ We continued this data analysis process by reading through each of the other participants’ transcripts one by one, on a case-by-case basis. We then approached the transcripts as a whole, making note of common themes that emerged across each of the participant’s responses. We reported these themes as the results of our study.^25^ Rather than using a numerical threshold for theme inclusion (eg, a minimum number of participants), we followed IPA’s emphasis on depth of meaning, recurrence across cases, and thematic coherence.^26^ Themes were identified through an iterative, case-by-case analysis of transcripts, followed by cross-case comparison. The final set of five themes was selected because they were both recurrent across multiple participants and analytically rich in describing the training needs for LSCO readiness.

Our research team consisted of two military physicians, two military medical students, and one Ph.D. curriculum researcher. As educators and military healthcare professionals, we recognized the influence of our own knowledge and perceptions on our interaction with our participants and our interpretation of the data, which is an inherent aspect of IPA.^27^ For example, we asked follow-up questions to the participants regarding aspects that we thought particularly salient to the research question during the interviews. We also read through the data with our literature review in mind, understanding the potential challenges of future LSCO as we analyzed the participants’ responses. While minor differences in interpretation emerged during the analysis process, a common occurrence in qualitative research, they were addressed through iterative discussions among the research team. All discrepancies were resolved by consensus.^28^

## RESULTS

**Figure 1:**
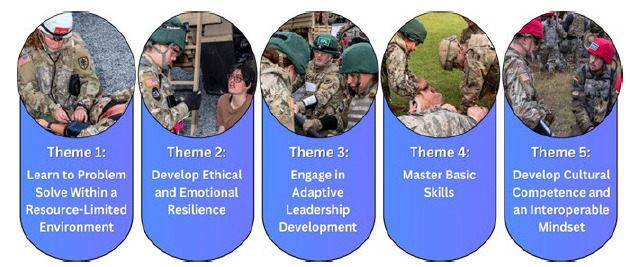
Five themes that emerged from interviews with 27 military medical faculty and one chaplain regarding medical education and training needs.

Five themes emerged from the data regarding medical students’ education and training needs: 1) Learn to problem solve within a resource-limited environment; 2) Develop ethical and emotional resilience; 3) Engage in adaptive leadership development; 4) Master basic skills; and 5) Develop cultural competence and an interoperable mindset. These themes and example images are presented in Figure 1.

### Theme 1: Learn to problem solve within a resource-limited environment

The participants first described the need to teach students to problem-solve within a resource-limited environment. To do so, students must develop a new way of thinking “way outside the box, to use, really to find whatever is available, to use whatever” (Participant [P] 9). This new thought process includes “being able to focus on a methodological or a logical way of thinking through novel problems that may not have existed before” (P2). Additionally, the participants described how students must learn to act decisively, as one noted,

. . . even if the decision is the wrong decision, that’s better than no decision at all . . . I don’t care if it’s perfect, I just care that you did something.” (P25)

Thinking ahead in their problem-solving is essential:

Everyone’s going to have a little bit of prolonged casualty care on the second- and third-order effects. You can do the tourniquet, but most people don’t think, ‘Okay, I’ve done intervention, what are the secondary and tertiary effects out of injuries?’ (P13)

This problem-solving skill set will be key when facing large numbers of patients with fewer resources during LSCO, where students may confront “very, very complicated questions about resource utilization and decisions on how you can do the greatest good for the greatest number” (P16). As a result, the participants emphasized the need to learn how to take care of more patients with fewer resources, encouraging students to

[I]dentify a problem, a medical-related problem, big or small, and then just like if you had resources, how would you propose to fix it? Just kind of encouraging those thought processes.” (P4)

The participants also described how medical students should be challenged to make the most of limited supplies. For example, understanding that

in deployed environments, you can ask for whatever you want, but . . . it might be a couple months before you get them, so you need to learn to figure out how to solve problems with what you’ve got. (P25).

### Theme 2: Develop ethical and emotional resilience

As a result of needing to do more with less, students must be prepared to navigate complex ethical issues that may lead to moral injury. One participant described how students will

have the tools and abilities within themselves to save someone, but the situation doesn’t allow it. And especially if it’s one of their people, their friends, one of their loved ones. (P15)

The scale of future conflicts may further intensify this risk, as another participant noted,

The numbers of casualties that we are going to see are going to be five, ten times larger than what we have seen now . . . Being exposed to all that kind of stuff exposes us to the potential for moral injury. (P25)

The participants also reflected on the need for students to build resilience for such hardships, with one stating,

What we need to do is protect them from that moral injury . . . So we’ve got to prepare them for the pain and suffering and arduousness of real war. (P9)

Another participant noted,

We need to prepare them for the fact that patients are going to die . . . because we’re not able to get them the supplies that they want.

And this participant echoed,

Preparing them for the adverse outcomes that they’re going to see, preparing them for all the crazy, wild things that we’re going to let them do . . . because they’re doing everything they can to save their patients. (P19)

Without regular practice in facing challenges and adversity, another participant added, “We’re not just automatically going to be good at facing challenges and adversity” (P25), as they emphasized the need for immersive preparation to help students confront and manage the emotional weight of real-world combat scenarios and the complex medical decision-making that will be required.

### Theme 3: Engage in adaptive leadership development

Given the complexities of LSCO, the participants described a pressing need for adaptive leadership training for future military physicians, as these physicians will lead teams in high-stress environments and must regularly adapt to new challenges.

We need to develop leaders that can deal with the complexities of technology and the weaponry that’s going to be there because it’s not going to be necessarily a bunch of blast victims right in front of us. It might be people much farther away. (P11)

Participant 13 likewise explained that as we move toward more disaggregated operations, individuals will need to “know how to lead teams and operate independently based on mission command type order sets…So they’re going to have to be more comfortable being uncomfortable” (P13). Given the potential high stress of LSCO, Participant 15 emphasized that medical students need to learn to lead with “coolness and calmness…you need to be able to command respect without demanding respect” (P15). Additionally, fostering teamwork was highlighted as essential to effective leadership: “If you’re not being a good teammate, you’re not being a good leader, physician, officer, and human” (P19). This sense of shared leadership was reinforced by the idea that leadership isn’t about a single authority, but rather about “empowering each team member based on their strengths, whether medics, nurses, or ambulance drivers” (P24).

In addition to leading and adapting under pressure during LSCO, our participants stressed that medical students must develop a flexible mindset.

You need a leader who can somehow balance the piece of being able to listen to feedback from people and take that as well as guiding the team toward an end. (P7)

Participant 15 described the need for flexibility in perspective as well, stating,

You have to be able to step back, see the big picture, understand the constraints of your environment, what is survivable, what is feasible, and what is reasonable. (P15)

Empathy and self-care were also identified as essential components of effective leadership. As one participant noted, “Leaders need to take time to care for themselves… you cannot give what you do not have” (P19).

### Theme 4: Master basic skills

In the midst of the new challenges that military physicians may encounter during LSCO, the participants emphasized the need for students to learn and remember fundamental lessons to “do the simple stuff in a really crappy environment” (P2). They highlighted that mastering the basics, such as applying a tourniquet, managing massive hemorrhage, managing an airway, and even knowing positioning techniques, is crucial for effective response. Underscoring the importance of a strong foundation in core knowledge and skills, one participant questioned,

If you can’t positively answer where you’re going to put a [needle decompression], how can you positively say where I’m going to put a chest tube or do a finger thoracostomy or at what phase of the treatment to do that? (P4)

Another noted the risk of skill decay in tactical medical skills, describing them as “very perishable,” which highlights the need for consistent practice in these essential areas (P4). Participant 16 also emphasized the importance of basic skills when practicing outside one’s specialty:

You’re not always going to have a full complement of specialists. And that it’s going to reinvigorate the art of the generalist and having a much broader, wider slice of the medical pie as part of your domain.

Finally, participants believed that teaching military history to students is an important part of their future preparation.

The only thing that we have right now is looking to the past, kind of studying that really closely and then figuring out the lessons learned there, like how we can apply those to the future. (P10)

Participant 8 echoed this perspective:

You can certainly go back in time, eons, and look at how it was dealt with through the Greeks or through the Romans . . . at many different levels.

### Theme 5: Develop cultural competence and an interoperable mindset

In preparing for future operations, participants highlighted the importance of developing cultural competence and an interoperable mindset. As one participant noted,

We need to learn other cultures, we need to learn intercultural relationships, we need to learn about other political systems, other social structures . . . especially as military leaders, we need to think wide.” (P20)

This cultural and political awareness is essential for effective communication and collaboration with partner nations. One faculty emphasized that understanding “their communication strategies and how they will work with other nations is also going to be...more important than it was in the last conflict.” (P21) Finally, in thinking about how to teach these skills, Participant 18 suggested that

having international students frequently integrated in the curriculum and within our exercises . . . working with partner forces throughout all four years [is key for military medical readiness].

## DISCUSSION

Our study’s results revealed five focus areas for preparing medical students for LSCO: 1) Learn to problem solve within a resource-limited environment; 2) Develop ethical and emotional resilience; 3) Engage in adaptive leadership development; 4) Master basic skills; and 5) Develop cultural competence and an interoperable mindset. Given the complex operational environments of LSCO, civilian and military medical students must learn to solve problems with few resources, lead diverse teams under pressure, and rely on their foundational medical training to make effective decisions in this type of wartime environment. These themes are relevant for both military and civilian medical students, since the scale of LSCO may necessitate civilian physician involvement in care delivery, making these themes broadly applicable across training environments. These themes can be operationalized by providing specific curricular strategies, such as embedding immersive simulation scenarios that replicate resource-constrained settings, introducing ethical case studies focused on moral injury, and integrating team-based leadership challenges modeled on LSCO conditions. We also recommend cross-institutional training initiatives that allow military and civilian students to learn side by side, enhancing interoperability and shared preparedness.

The anticipated resource constraints in future large-scale conflicts highlight the necessity of equipping healthcare teams with the knowledge and skills to make complex decisions about resource allocation and to foster innovation in addressing novel challenges.^29^ Innovation in non-traditional patient care settings has long been a hallmark of military medicine, with significant advancements translating to civilian medical care over the past two decades.^30^ Similarly, civilian emergency response frameworks have informed military medical strategies, emphasizing the reciprocal benefits of military-civilian collaboration.^31^ While we acknowledge that civilian medical students do not currently require the same degree of preparation as their military counterparts, we believe that our findings offer a forward-looking framework for potential curricular adaptation should civilian systems be more directly involved in future LSCO, as they are predicted to be.

Because both civilian and military medical students are in an early, formative stage of development, we advocate for a developmentally appropriate, scaffolded approach that introduces foundational concepts—such as ethical reasoning, resource-conscious thinking, and adaptive leadership mindsets—early in training and builds progressively across the undergraduate medical education continuum. Specific skills such as tactical triage would be introduced through guided simulation and interdisciplinary exercises, rather than full clinical implementation during early training years. Overall, these recommendations are not intended to duplicate existing curricula but to intentionally adapt and expand them to address the escalating demands of LSCO.

As our participants described, effective problem-solving and innovation require a strong foundation of basic knowledge, and skills.^32^

This foundation aligns closely with the concept of adaptive expertise, a critical competency in medical education. Adaptive expertise is defined as “the effective application of existing knowledge and skills to create innovative solutions for tasks or problems that are novel to the expert.”^33^ Unlike routine expertise, which focuses on mastering procedures and algorithms, adaptive expertise involves a deeper understanding of underlying principles and the flexibility to apply that knowledge in new and unexpected contexts.^34^ Key attributes of adaptive expertise—flexibility, problem-solving, innovation, and comfort with uncertainty—align closely with the enhanced roles and attributes required of modern healthcare leaders, both military and civilian.^34^ These characteristics also resonate with the themes highlighted by our participants, underscoring their importance in preparing healthcare professionals for the complex and unpredictable challenges of future crisis environments.

Educational strategies that promote the development of adaptive expertise often emphasize conceptual knowledge to prepare learners for future learning, which refers to “the ability to learn new information, effectively utilize resources, and develop new procedures to support learning and problem-solving in practice.”^35^ Teaching methods that foster preparing learners for future learning include offering diverse experiential learning opportunities, allowing students to face challenges and occasionally fail, encouraging self-reflection, and providing timely direct instruction or feedback.^36^, ^37^ Simulation-based education, like the training environment in our study, serves as an excellent experiential learning tool. It enables learners to make and learn from mistakes in a safe environment, fostering adaptability, innovation, and a problem-solving mindset.^38^

Our study likewise emphasized the need for adaptive leadership training to prepare students for the uncertainties of LSCO and large-scale disasters, which will result in complex leadership challenges. This leadership training, for example, might focus on navigating issues such as moral injury and expectant casualty care due to the extensive numbers of casualties expected in such scenarios. This training should begin early (ie, during medical school) and continue throughout a physician’s career to ensure comprehensive preparedness for crisis leadership. By embedding leadership scenarios into early and ongoing training, medical students could practice managing complex team dynamics, implementing tactical triage, and working effectively in multidisciplinary, high-stakes environments. Additionally, simulations could be used to develop resilience, encouraging future physicians to recognize the emotional impact of crisis care and providing strategies for coping with the moral and psychological stressors unique to LSCO and disaster response.^39^

During LSCO, patient care prioritization and treatment protocols might shift in crisis scenarios, from life-saving interventions to symptom management and ethical end-of-life care.^40^ Our participants identified moral injury as a major challenge for healthcare professionals when making difficult decisions about resource allocation. The COVID-19 pandemic underscored the severe impact of moral injury on physicians’ well-being,^41^ and further studies highlight both military and civilian healthcare teams’ vulnerability to moral injury in crisis settings, with potential negative effects on mental health, decision-making, and resilience.^10^,^11^,^42^ Large-scale combat operations may introduce unique ethical tensions, such as dual loyalty and battlefield triage, which heighten moral distress and the risk of moral injury.^43^,^44^,^45^

Preparing healthcare teams for these ethical complexities is critical for both mission readiness and effective disaster response.^43^,^44^,^45^,^46^,^47^ Addressing these challenges requires building resilience by recognizing, anticipating, and mitigating moral injury through support strategies for individuals and teams.^48^ Educating medical students about ethical decision-making frameworks and moral injury can increase self-awareness and normalize the concept within both military and civilian healthcare.^49^

Ethical frameworks provide principles for navigating morally complex situations in medical practice. Their effectiveness is well-documented in hospital settings^50^ and civilian emergency response.^47^,^51^,^52^ However, adapting them to large-scale crisis care requires addressing challenges like dual loyalty, constrained medical resources, and the inability to determine patient preferences in chaotic environments.^53^,^54^ Thus, specialized ethical frameworks and targeted education tailored to both armed conflict and civilian disaster response are essential for preparing healthcare teams.^53^,^54^ Additionally, clear guidelines for expectant casualty care from organizations like the Joint Trauma System, alongside training in these clinical practice guidelines, may reduce moral injury.^11^

In preparing for future operations, participants emphasized the importance of fostering cultural competence and an interoperable mindset. Military healthcare teams are increasingly required to collaborate with medical personnel from other countries as part of multinational forces. This collaboration is also seen between healthcare systems and agencies within the US who collaborate to manage disaster responses. The challenges of wartime collaboration and healthcare delivery in such settings are well-documented.^55^ While the benefits of international medical cooperation are widely acknowledged (RAND Corporation; North Atlantic Treaty Organization), there is a pressing need for innovative education and training programs within both civilian and military medical education that enhance interoperability.^56^,^57^ Such efforts can improve healthcare delivery, strengthen global alliances, and support more effective humanitarian initiatives worldwide.^58^

This emphasis on interoperability aligns with calls from the international education community, particularly after the COVID-19 pandemic, to define and integrate international leadership competencies into medical education.^59^ These competencies promote a collaborative approach to addressing global challenges and align with the skills identified by our faculty. Key competencies include “international specialized knowledge,” such as understanding differences among organizational and educational systems, and “integrated leadership skills,” including cultural competence, humility, respect for others’ customs and languages, empathy, and openness to diverse perspectives.^59^

Finally, a recently emerging concept, the “duty of mind,” emphasizes a physician’s responsibility to maintain ethical decision-making under crisis-induced stress. Failure to do so can lead to biased resource allocation and suboptimal decisions.^60^ While experience helps mitigate stress-related impacts, many healthcare professionals lack direct exposure to crisis scenarios, particularly in the current inter-war and inter-disaster periods.^60^ Medical education plays a crucial role in bridging this gap and preparing professionals for expanded scopes of practice in evolving conflict and disaster scenarios.^13^,^61^

## LIMITATIONS

Our participants consisted of faculty members teaching at our university’s capstone Medical Field Practicum who volunteered their time and expertise. Because these faculty had deployment experience only to Iraq or Afghanistan, they could only speculate about what future large-scale combat operations might consist of. In addition, our study lacks quantitative outcome data measuring the impact of various training approaches on medical student readiness. Future research should develop and evaluate curricula centered around the themes described in our study to quantify their utility for training future military physicians for LSCO. Finally, our participants were all military medical educators, not civilian medical educators. Any references to civilian training are framed as potential areas for exploration or cross-contextual relevance, rather than definitive claims. This distinction should help clarify the scope and positioning of the participants’ insights. Future research studies should explore civilian medical educators’ perception of needed educational training for LSCO as well, to offer a more comprehensive viewpoint and evaluation of training needs.

## CONCLUSION

This study identified five core competencies—problem-solving in resource-limited environments; ethical and emotional resilience; adaptive leadership; mastery of core medical skills; and cultural competence with an interoperable mindset—that experienced military educators view as essential for preparing military and civilian medical students for large-scale combat operations. While many of these competencies overlap with those emphasized in disaster and mass casualty response, large-scale combat operations introduce distinct challenges, such as prolonged austere care, contested environments, and complex ethical dilemmas that require focused educational attention.

Our findings suggest that early, developmentally appropriate exposure to these concepts in undergraduate medical education can lay the groundwork for future crisis readiness. While we do not propose that medical students independently lead tactical triage or complex field care, foundational training in decision-making under pressure, leadership mindsets, and ethical reflection can support progressive competence across the medical education continuum.

Although our study’s participants consisted of military medical faculty, the themes identified are increasingly relevant for civilian physicians as well. Future LSCO may overwhelm military medical systems, requiring civilian clinicians to care for patients in similar high-stakes conditions. Integrating these competencies into civilian training—through case-based learning, simulation, and interprofessional exercises—can strengthen cross-sector preparedness.

These findings offer an evidence-informed foundation for future curriculum development and call for expanded collaboration between military and civilian educators to prepare the next generation of physicians for the evolving demands of conflict and crisis medicine.

## Supplementary Information
