# Supplementary material for: Large-Scale Combat Operation Education and Training Needs: Implications for Military and Civilian Medical Education
Source: West J Emerg Med. 2025 Sep 1;26(5):1144–53. doi: 10.5811/westjem.43557 (PMC12591639; doi:10.5811/westjem.43557)
Supplement: Supplementary file 1 [file wjem-26-1144-s001.docx]

**Appendix A. Participant Demographics**

**Category Number of Participants**

| **Rank (higher number corresponds to higher ranking military officer)** |  |
| --- | --- |
| 06 | 6 |
| 05 | 14 |
| 04 | 6 |
| 03 | 1 |
| **Specialty** |  |
| Trauma Critical Care | 2 |
| Preventative Medicine | 2 |
| Anesthesiology | 2 |
| Occupational Medicine | 1 |
| Pediatrics | 1 |
| Emergency Medicine | 10 |
| Psychiatry | 1 |
| Family Medicine | 6 |
| Aerospace Medicine | 1 |
| Chaplain | 1 |
| **Branch of Service** |  |
| Army | 10 |
| Navy | 8 |
| Air Force | 7 |
| United States Public Health Service | 1 |
| Army National Guard | 1 |
| **Status** |  |
| Active-duty | 24 |
| Reservist | 3 |
